# Epidemiological characteristics and quarantine assessment of imported international COVID-19 cases, March to December 2020, Chengdu, China

**DOI:** 10.1038/s41598-022-20712-8

**Published:** 2022-12-07

**Authors:** Wenqiang Zhang, Yong Yue, Min Hu, Changhui Du, Cheng Wang, Xiaoli Tuo, Xiaoman Jiang, Shuangfeng Fan, Zhenhua Chen, Heng Chen, Xian Liang, Rongsheng Luan

**Affiliations:** 1 Chengdu Workstation for Emerging Infectious Disease Control and Prevention, Chinese Academy of Medical Sciences, Chengdu, 610041 Sichuan China; 2 Department of Epidemiology and Health Statistics, West China School of Public Health and West China Fourth Hospital, Sichuan University, Chengdu, 610041 Sichuan China; 3 Chengdu Center for Disease Control and Prevention, Chengdu, 610041 Sichuan China

**Keywords:** Preventive medicine, Epidemiology, Viral infection

## Abstract

International flights have accelerated the global spread of Coronavirus Disease 2019 (COVID-19). Determination of the optimal quarantine period for international travelers is crucial to prevent the local spread caused by imported COVID-19 cases. We performed a retrospective epidemiological study using 491 imported COVID-19 cases in Chengdu, China, to describe the characteristic of the cases and estimate the time from arrival to confirmation for international travelers using nonparametric survival methods. Among the 491 imported COVID-19 cases, 194 (39.5%) were asymptomatic infections. The mean age was 35.6 years (SD = 12.1 years) and 83.3% were men. The majority (74.1%) were screened positive for SARS-CoV-2, conducted by Chengdu Customs District, the People’s Republic of China. Asymptomatic cases were younger than presymptomatic or symptomatic cases (*P* < 0.01). The daily number of imported COVID-19 cases displayed jagged changes. 95% of COVID-19 cases were confirmed by PT-PCR within 14 days (95% CI 13–15) after arriving in Chengdu. A 14-day quarantine measure can ensure non-infection among international travelers with a 95% probability. Policymakers may consider an extension of the quarantine period to minimize the negative consequences of the COVID-19 confinement and prevent the international spread of COVID-19. Nevertheless, the government should consider the balance between COVID-19 and socioeconomic development, which may cause more serious social and health crises.

## Introduction

Coronavirus Disease 2019 (COVID-19), caused by the severe acute respiratory syndrome coronavirus 2 (SARS-CoV-2), has affected hundreds of millions of people and caused millions of deaths globally^1^. International flights provide the opportunity for the global spread of COVID-19^2^. In Africa, air traffic is significantly associated with COVID-19 morbidity and mortality, and plays an important role in the geographical spread of the disease^3^. Travel restriction and travel quarantine have helped mitigate the international spread of COVID-19^2,4,5^.

The quarantine duration was determined by the incubation period, the time from exposure to an infectious disease to the onset of symptoms. Theoretically, it’s preferable to quarantine international travelers for a longer period than the maximum incubation period since the person-to-person transmission prior to symptom onset^6–9^. The estimated length of the incubation period of COVID-19 varies across the disease severity, model methods, and sample sizes^10–21^. Thus, the optimal quarantine duration for COVID-19 needs to be assessed using the previously reported incubation period.

Furthermore, a quarantine period varies all over the world. It is unclear whether the 14-day quarantine is sufficient to protect against the international spread of COVID-19. There is evidence that the maximum interval between entering mainland China and diagnosis for imported COVID-19 cases is 23 days^22^. However, the effectiveness of 14-day quarantine and Customs screening at early detection of international travelers remains poorly understood. Therefore, in this study, we provide direct estimates of the optimal quarantine period for international travelers, based on the interval between the date of arrival and the date of confirmation of imported COVID-19 cases in Chengdu, China. We also evaluate the effectiveness of Customs screening at detecting international travelers infected with SARS-CoV-2, which has significant implications for quarantine and isolation of international travelers in the future.

## Results

### Demographic characteristics of COVID-19 cases

Among the 491 imported COVID-19 cases (Table 1), 194 cases were asymptomatic (39.5%), 114 cases were presymptomatic (23.2%), 183 cases were symptomatic (37.3%). The mean age of the cases was 35.6 years (SD = 12.1). 409 cases were men (83.3%), 82 cases were women (16.7%). Most cases were workers (189, 38.5%), from Asia (266, 54.2%). More than half (283, 57.6%) came to Chengdu, China in autumn (September to November). The majority (364, 74.1%) of cases were screened positive for SARS-CoV-2, conducted by Chengdu Customs District, the People’s Republic of China. Compared with presymptomatic or symptomatic cases, asymptomatic cases had a lower mean age (*P* < 0.001). There was no sex or occupation difference across different stages of infections (*P* > 0.05), whereas an imported continent difference and seasonal difference were observed (*P* = 0.007, Fisher’s exact test; *P* = 0.025). The proportion of cases screened positive by Customs quarantine was slightly different (*P* = 0.043), while pairwise comparisons between the stages of infections showed no statistical difference (*P* > 0.05).

**Table 1:** Demographic characteristics of imported COVID-19 cases in Chengdu, China.

	Total (N = 491)	Asymptomatic (N = 194)	Presymptomatic (N = 114)	Symptomatic (N = 183)	*P* value
Age, years	35.6 ± 12.1	32.3 ± 10.9^†^	36.9 ± 13.2	38.3 ± 11.7	< 0.001
**Sex**					0.054
Men	409 (83.3)	152 (78.4)	100 (87.7)	157 (85.8)	
Women	82 (16.7)	42 (21.6)	14 (12.3)	26 (14.2)	
**Occupation**					0.215
Worker	189 (38.5)	72 (37.1)	42 (36.8)	75 (41.0)	
Cadre or staff	65 (13.2)	29 (14.9)	16 (14.0)	20 (10.9)	
Student or teacher	63 (12.8)	27 (13.9)	9 (7.9)	27 (14.8)	
Commercial service	62 (12.6)	27 (13.9)	16 (14.0)	19 (10.4)	
Housework or unemployment	40 (8.1)	16 (8.2)	9 (7.9)	15 (8.2)	
Catering service	34 (6.9)	15 (7.7)	6 (5.3)	13 (7.1)	
Other	38 (7.7)	8 (4.1)	16 (14.0)	14 (7.7)	
**Imported continent**					0.007^‡^
Asia	266 (54.2)	124 (63.9)	59 (51.8)	83 (45.4)	
Africa	166 (33.8)	50 (25.8)	45 (39.5)	71 (38.8)	
Europe	38 (7.7)	15 (7.7)	6 (5.3)	17 (9.3)	
Americas	21 (4.3)	5 (2.6)	4 (3.5)	12 (6.6)	
**Season at the entry**					0.025
Spring (March–May)	53 (10.8)	15 (7.7)	11 (9.6)	27 (14.8)	
Summer (June–August)	119 (24.2)	40 (20.6)	30 (26.3)	49 (26.8)	
Fall (September–November)	283 (57.6)	129 (66.5)	60 (52.6)	94 (51.4)	
Winter (December)	36 (7.3)	10 (5.2)	13 (11.4)	13 (7.1)	
**Customs quarantine**					0.043
Positive	364 (74.1)	150 (77.3)	90 (78.9)	124 (67.8)	
Negative	127 (25.9)	44 (22.7)	24 (21.1)	59 (32.2)	

Data are mean ± standard deviation or n (%).

^†^Compared with presymptomatic or symptomatic cases, *P* < 0.01, adjusted using the Bonferroni correction.

^‡^Fisher’s exact test.

### Time to confirmation

**Figure 1:**
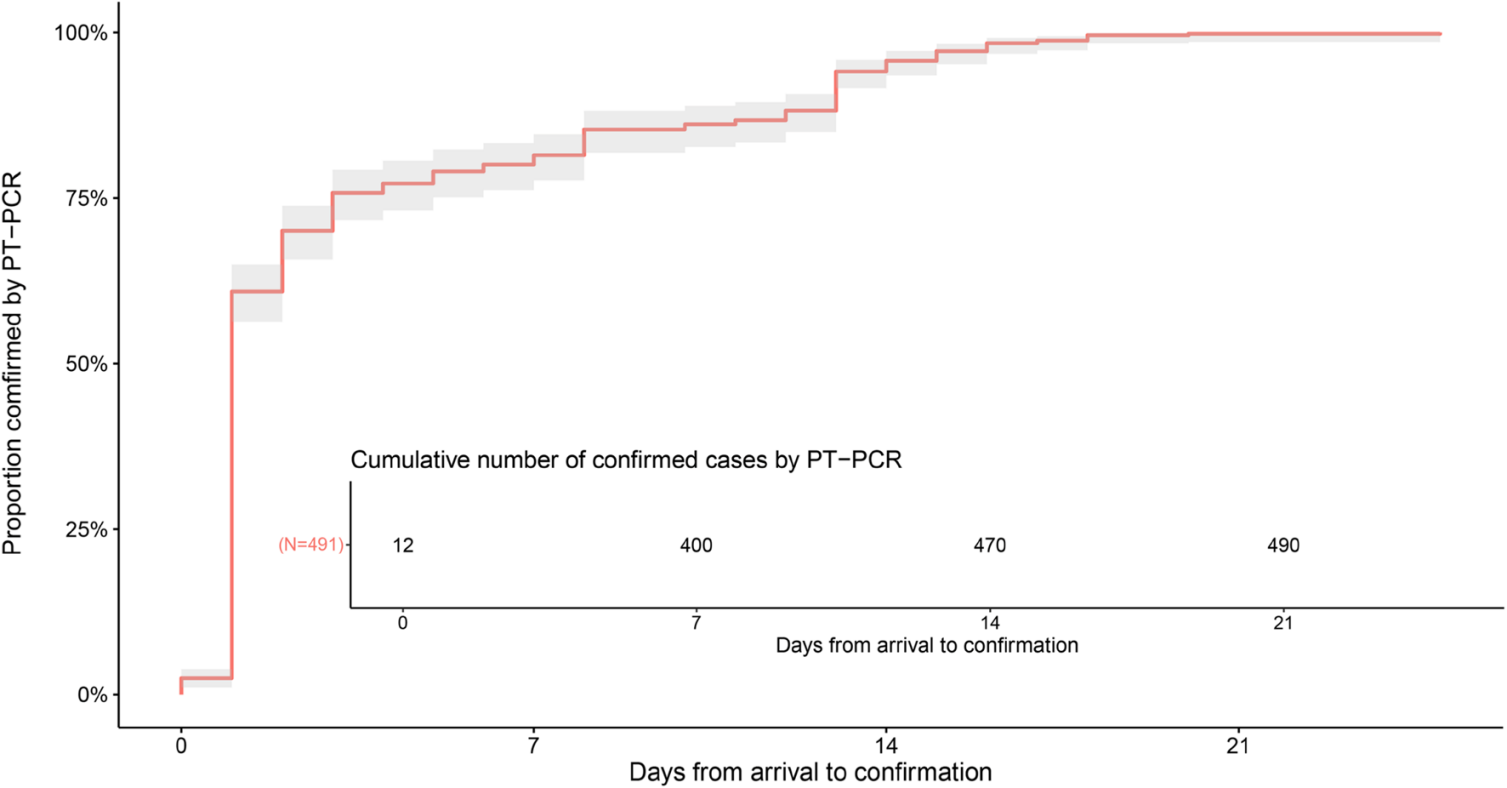
Time between arrival in Chengdu and SARS-CoV-2 confirmation. We estimated that 95% of cases were confirmed by PT-PCR within 14 days (95% CI 13–15) after arriving in Chengdu.

**Figure 2:**
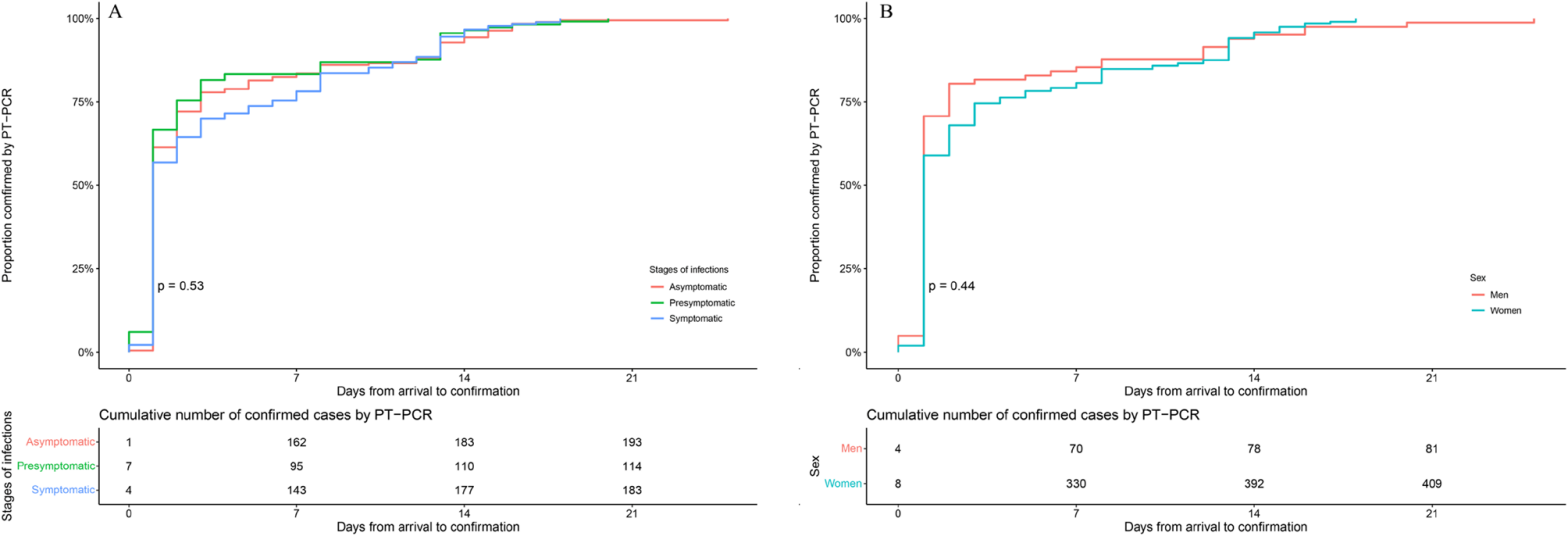
Time between arrival in Chengdu and SARS-CoV-2 confirmation by stages of infections (**A**), sex (**B**). Panel A shows estimates of the proportion of cases at different stages who are confirmed by PT-PCR, according to the number of days after arriving in Chengdu. We estimated that 95% of asymptomatic cases were confirmed by PT-PCR within 15 days (95% CI 13–16) after arrival, 95% of presymptomatic cases were confirmed by PT-PCR within 13 days (13–13) after arrival, 95% of symptomatic cases were confirmed by PT-PCR within 14 days (13–17) after arrival. Panel B shows estimates of the proportion of cases for different sexes who are confirmed by PT-PCR, according to the number of days after arriving in Chengdu. We estimated that 95% of male cases were confirmed by PT-PCR within 14 days (95% CI 13–15) after arrival, 95% of female cases were confirmed by PT-PCR within 14 days (12–16) after arrival. The upper confidence limit for female cases and presymptomatic cases are not available and estimated using the 95th quantile and corresponding lower confidence limit.

The cumulative distribution of time to confirmation, defined as the time between the date of arriving in Chengdu and the date of confirmation of COVID-19 by PT-PCR, is shown in Fig. 1. Among 491 cases, 95% were confirmed by PT-PCR within 14 days (95% CI 13–15) after arriving in Chengdu. For different stages of infections (Fig. 2A), 95% of asymptomatic cases were confirmed by PT-PCR within 15 days (13–16) after arrival, 95% of presymptomatic cases were confirmed by PT-PCR within 13 days (13–13) after arrival, 95% of symptomatic cases were confirmed by PT-PCR within 14 days (13–17) after arrival. Furthermore, 95% of male cases were confirmed by PT-PCR within 14 days (13–15) after arrival, 95% of female cases were confirmed by PT-PCR within 14 days (12–16) after arrival (Fig. 2B).

### Factors associated with time to confirmation

Table 2 shows the results of the univariable and multivariable regression model analyses for factors associated with the time to confirmation of imported COVID-19 cases. The univariable analysis of confirmation time shows that symptomatic patients had a short time to confirmation compared to asymptomatic infections (OR = 1.62; 95%CI = 1.03–2.57). This association was further supported by multivariable analyses and was not affected by potential confounding factors (OR = 1.87; 95%CI = 1.15–3.07). We did not observe any additional associated factors.

**Table 2:** Logistic regression analyses for factors associated with time to confirmation of imported COVID-19 cases in Chengdu, China.

	Univariable model	Multivariable model
**Age**		
< 35	1.00 (reference)	1.00 (reference)
≥ 35	0.90 (0.60–1.34)	0.78 (0.49–1.24)
**Sex**		
Men	1.41 (0.81–2.57)	1.22 (0.65–2.36)
Women	1.00 (reference)	1.00 (reference)
**Occupation**		
Worker	1.15 (0.76–1.73)	1.38 (0.83–2.30)
Nonworker	1.00 (reference)	1.00 (reference)
**Imported continent**		
Asia	1.84 (1.16–2.98)	2.02 (1.23–3.41)
Africa	1.00 (reference)	1.00 (reference)
Europe	1.56 (0.66–3.46)	2.14 (0.82–5.36)
Americas	2.18 (0.77–5.71)	2.65 (0.87–7.66)
**Season at the entry**		
Spring (March–May)	1.00 (reference)	1.00 (reference)
Summer (June–August)	1.58 (0.73–3.66)	1.63 (0.66–4.24)
Fall (September–November)	1.55 (0.77–3.41)	1.67 (0.73–4.09)
Winter (December)	1.65 (0.60–4.56)	2.23 (0.76–6.70)
**Stages of infections**		
Asymptomatic	1.00 (reference)	1.00 (reference)
Presymptomatic	0.91 (0.51–1.58)	1.02 (0.56–1.82)
Symptomatic	1.62 (1.03–2.57)	1.87 (1.15–3.07)

Data are presented as odds ratios (95% confidence interval).

## Discussion

This analysis of imported COVID-19 cases in Chengdu, China, provides insight into formulating a rationale for determining the optimal quarantine period for international travelers. The values estimated provide the evidentiary foundation for containing the international spread of COVID-19. Analyses of the interval between the date of arrival and the date of confirmation of imported COVID-19 cases revealed that at least 5% of incubation periods could be longer than 14 days. These results paint a positive picture of the impact of obligatory household quarantine for 14 days in Chengdu. Public health authorities may consider a slight extension of the quarantine period to deal with uncertainty in the incubation periods.

All ages of the population are susceptible to SARS-CoV-2 infection, younger adults who contracted SARS-CoV-2 are more likely to be asymptomatic or have mild diseases^23–25^. We found that asymptomatic cases were younger than presymptomatic or symptomatic cases. The age-dependent T-cell and B-cell dysfunction and differential expression of inflammatory genes could lead to immune dysregulation and induce more prolonged proinflammatory responses, potentially contributing to poor outcomes^26–28^.

This work further supports a high prevalence of asymptomatic cases in SARS-CoV-2 infections^22,29,30^. 39.5% of international travelers infected with SARS-CoV-2 remains asymptomatic, which is lower than the range reported for SARS-CoV-2 (40–45%)^30^. Asymptomatic international travelers will be missed by symptom-based surveillance^31^, even if screened for SARS-CoV-2. Furthermore, only 74.1% of imported COVID-19 cases were timely found by Customs quarantine. It is crucial to implement the travel quarantine for international travelers during the COVID-19 pandemic.

Our estimation of the 95th quantile of the interval between the date of arrival and the date of confirmation, 14 days, supports the adopted mandatory quarantine period of 14 days in China^19^. Of note, we estimated that about 5.0% of imported COVID-19 cases would not have a positive SARS-CoV-2 RT-PCR array until 14 days after arriving in Chengdu. Similarly, previous work had suggested that at least 5% of cases would take 14 days or more to develop symptoms^19–21^. In particular, asymptomatic cases can transmit SARS-CoV-2 to others for longer than 14 days^30^. It has appeared several COVID-19 outbreaks in China due to international imported COVID-19 cases^32^. Consequently, the 14-day household quarantine was recommended to international travelers discharged from a designated centralized quarantine site for 14 days in Chengdu.

Besides, we found that symptomatic patients had a short time to confirmation of SARS-CoV-2 compared to asymptomatic infections, which be accountable for that the symptomatic individuals had a significantly higher peak mean viral load than the asymptomatic SARS-CoV-2 infections^33^. A prior study also found nationality, the interval between entering and detection of positive results, the results of initial nucleic acid detection were critical factors for time to confirmation^22^. Policy makers should take into account these important factors.

Our study has several limitations. First, international travelers arriving in Chengdu might be a biased sample due to the airline control measures implemented by the Civil Aviation Administration of China. Second, we could not estimate the incubation period distribution of imported COVID-19 cases since the possible exposure time of imported COVID-19 cases was not available. Third, a minority of international travelers who arrived in December 2020 received COVID-19 vaccines, so we could not evaluate the impact of the vaccines. Although vaccination is effective and safe against COVID-19^34,35^, the vaccinated population may contract SARS-CoV-2 and infect others^36–38^. Moreover, we could not assess the true transmissibility of imported cases with an extended incubation period, longer than 14 days. A longitudinal study should be carried out to investigate the infectious period of COVID-19 or how long infected individuals remain infectious to others, which is associated with release from quarantine. Finally, the virus mutation may markedly change the transmissivity and severity of SARS-Cov-2, causing the quarantine policy adjustment, and future studies should take into consideration different SARS-Cov-2 variants.

## Conclusions

The high prevalence of asymptomatic infection and the long incubation period have made it challenging to contain the COVID-19 pandemic. To ensure non-infection with a 95% probability, public health authorities should quarantine international travelers for more than 14 days. Also, policymakers may consider an obligatory household quarantine or household health monitoring after a 14-day designated centralized quarantine, to minimize the negative consequences of the COVID-19 confinement and prevent the international spread of COVID-19. Nevertheless, the government should consider the balance between COVID-19 and socioeconomic development, which may cause more serious social and health crises.

## Methods

### Source of data

A retrospective observational study of the imported laboratory-confirmed COVID-19 cases from March 2020 to December 2020 was performed. All international travelers, arriving in Chengdu, should be screened for SARS-CoV-2, shortly conducted by Chengdu Customs District, the People’s Republic of China. Confirmations of COVID-19 cases were done at the Chengdu Center for Disease Control and Prevention, using reverse transcription polymerase chain reaction (RT-PCR) assays targeting two different regions of the RdRp gene in SARS-CoV-2. All methods were carried out in accordance with relevant guidelines. All experiments were carried out in the Laboratory of Chengdu Center for Disease Control and Prevention. Regardless of symptoms, all international travelers should comply with a centralized isolated medical quarantine for 14 days and household quarantine for 14 days in Chengdu. Basic demographic information and time from arrival to confirmation by RT-PCR were recorded for all confirmed cases. We excluded 2 imported COVID-19 cases who received a centralized isolated medical quarantine in the other city, subsequently arrived in Chengdu, and were confirmed by RT-PCR. Hence, we included 491 cases in the study.

### Case definitions

COVID-19 cases were divided into asymptomatic, presymptomatic, and symptomatic cases. Asymptomatic cases were defined as being confirmed by RT-PCR without any symptoms whatsoever for the duration of infection (e.g., fever, cough, anosmia, and lung changes). Presymptomatic cases were defined as being confirmed by RT-PCR, prior to the onset of symptoms. Symptomatic cases were defined as being confirmed by RT-PCR and the development of symptoms.

### Time to confirmation

In this study, “time to confirmation” was defined as the interval in days between the date of arriving in Chengdu and the date of confirmation of COVID-19 by PT-PCR.

### Statistical analysis

Age was present as mean with standard deviation (SD), which was subject to normal distribution. We performed the Shapiro–Wilk test of normality and computed Levene’s test for homogeneity of variance across the stages of infections (package = “car”, version = “3.0–11”). In this study, the variance was tested to be not equal. We used an approximate method of Welch^39^ to compare age according to the stages of infections, which generalizes the commonly known 2-sample Welch test to the case of arbitrarily many samples. Pairwise comparisons between the stages of infections were calculated with the Bonferroni corrections for multiple testing (package = “stats”, version = “4.1.0”). Sex, occupation, imported continent, season at the entry, Customs quarantine were presented as absolute numbers with the percentage. Chi-square tests and Fisher’s exact tests were used to compare the difference according to the stages of infections (package = “gmodels”, version = “2.18.1”; package = “rcompanion”, version = “2.4.1”). Time between arrival and confirmation was estimated by the use of nonparametric survival methods. The 95th quantile of time to be confirmation and corresponding 95% confidence interval (*CI*) were computed (package = “survival”, version = “3.2–11”; package = “survminer”, version = “0.4.9”). A univariable logistic regression model and a multivariable logistic regression model were used to estimate the odds ratios and 95% confidence interval to explore the factors associated with time to confirmation of imported COVID-19 cases in Chengdu, China (package = “stats”, version = “4.1.0”). The outcome was defined as whether the time to confirmation was below one day, that is, immediately screened positive for SARS-CoV-2, conducted by Chengdu Customs District, the People’s Republic of China. All analyses were performed using R version 4.0.4 (R Foundation for Statistical Computing). A *P* value (*P* < 0.05) was used to define statistical significance.

### Ethical approval

Data collection was determined by the Article 7, 12, 48, Law of the People’s Republic of China on Prevention and Treatment of Infectious Diseases to be part of the epidemiological investigation of a notifiable infectious disease and therefore the individual informed consent was waived. Partial data was available from the official website of Chengdu Municipal Health Commission (http://cdwjw.chengdu.gov.cn/cdwjw/c135632/yqbd.shtml). Paragraph 1 of Article 7: Center for Disease Control and Prevention at all levels shall undertake infectious disease monitoring, prediction, epidemiological investigation, epidemic reporting, and other prevention and control work. Paragraph 1 of Article 12: All units and individuals within the territory of the People’s Republic of China must accept the investigation, inspection, sample collection, isolation treatment and other prevention and control measures associated with infectious diseases by Center for Disease Control and Prevention and medical institutions, and truthfully provide relevant information. Center for Disease Control and Prevention and medical institutions shall not disclose relevant information and materials involving personal privacy. Paragraph 1 of Article 48: When an epidemic of infectious diseases occurs, Center for Disease Control and Prevention and other professional and technical institutions related to infectious diseases designated by the Administrative Department of Health of the People's Government at or above the provincial level may enter the epidemic focus and epidemic area of infectious diseases for investigation, sample collection, technical analysis and testing.

## Supplementary Information

Supplementary Information.

## Data Availability

All data are available in the supplementary material. All codes are available by emailing Wenqiang Zhang (zwqscu@126.com) on reasonable request.
